# The realized efficacy of indoor residual spraying campaigns falls quickly below the recommended WHO threshold when coverage, pace of spraying and residual efficacy on different wall types are considered

**DOI:** 10.1371/journal.pone.0272655

**Published:** 2022-10-03

**Authors:** Lucia Fernández Montoya, Mara Máquina, Helena Martí-Soler, Ellie Sherrard-Smith, Celso Alafo, Mercy Opiyo, Kiba Comiche, Beatriz Galatas, Silvie Huijben, Lizette L. Koekemoer, Shüné V. Oliver, Francois Maartens, Dulcisaria Marrenjo, Nelson Cuamba, Pedro Aide, Francisco Saúte, Krijn P. Paaijmans

**Affiliations:** 1 Centro de Investigação em Saúde de Manhiça (CISM), Manhiça, Mozambique; 2 ISGlobal, Barcelona, Spain; 3 MRC Centre for Global Infectious Disease Analysis, Imperial College London, London, United Kingdom; 4 Center for Evolution and Medicine, School of Life Sciences, Arizona State University, Tempe, Arizona, United States of America; 5 Simon A. Levin Mathematical, Computational and Modeling Sciences Center, Arizona State University, Tempe, Arizona, United States of America; 6 Wits Research Institute for Malaria, School of Pathology, Faculty of Health Sciences, Johannesburg, South Africa; 7 Centre for Emerging Zoonotic and Parasitic Diseases, National Institute for Communicable Diseases of the National Health Laboratory Service, Johannesburg, South Africa; 8 Good Bye Malaria, Johannesburg, South Africa; 9 National Malaria Control Programme, Ministry of Health, Maputo, Mozambique; 10 PMI VectorLink Project, Abt Associates Inc., Maputo, Mozambique; 11 National Institute of Health, Ministry of Health, Maputo, Mozambique; 12 The Biodesign Center for Immunotherapy, Vaccines and Virotherapy, Arizona State University, Tempe, AZ, United States of America; George Mason University, UNITED STATES

## Abstract

Indoor residual spraying (IRS) has been and remains an important malaria control intervention in southern Mozambique, South Africa and Eswatini. A better understanding of the effectiveness of IRS campaigns is critical to guide future elimination efforts. We analyze the three IRS campaigns conducted during a malaria elimination demonstration project in southern Mozambique, the “*Magude project*”, and propose a new method to calculate the efficacy of IRS campaigns adjusting for IRS coverage, pace of house spraying and IRS residual efficacy on different wall types. *Anopheles funestus sensu lato* (*s*.*l*.) and *An*. *gambiae s*.*l*. were susceptible to pirimiphos-methyl and DDT. *Anopheles funestus s*.*l*. was resistant to pyrethroids, with 24h post-exposure mortality being lower for *An*. *funestus sensu stricto* (*s*.*s*.) than for *An*. *parensis* (collected indoors). The percentage of structures sprayed was above 90% and percentage of people covered above 86% in all three IRS campaigns. The percentage of households sprayed was above 83% in 2015 and 2016, but not assessed in 2017. Mosquito mortality 24h post-exposure stayed above 80% for 196 days after the 2016 IRS campaign and 222 days after the 2017 campaign and was 1.5 months longer on mud walls than on cement walls. This was extended by up to two months when 120h post-exposure mortality was considered. The district-level realized IRS efficacy was 113 days after the 2016 campaign. While the coverage of IRS campaigns in Magude were high, IRS protection did not remain optimal for the entire high malaria transmissions season. The use of a longer-lasting IRS product could have further supported the interruption of malaria transmission in the district. To better estimate the protection afforded by IRS campaigns, National Malaria Control Programs and partners are encouraged to adjust the calculation of IRS efficacy for IRS coverage, pace of house spraying during the campaign and IRS efficacy on different wall types combined with wall type distribution in the sprayed area.

## Introduction

Indoor residual spraying (IRS) has been and remains a cornerstone intervention in malaria elimination efforts in southern Mozambique [1, 2]. Historically, it has been the core intervention in several initiatives that aimed to eliminate malaria in the southern part of the country, South Africa and Eswatini [3]. Between 1960 and 1969, malaria elimination was attempted using IRS with DDT [4]. During the Lubombo Spatial Development Initiative (LSDI, 2000–2011), IRS with bendiocarb was used in combination with treatment with artemisinin-based combination therapy (ACT) [3]. Since 2015, the Mozambique, South Africa, Eswatini (MOSASWA) initiative has been implementing IRS in Maputo Province, first using Actellic® 300CS (Syngenta Crop Protection AG, Switzerland) and DDT, and later SumiShield^TM^ 50WG (Sumitomo Chemical Company Ltd., Japan) and Fludora® Fusion (Bayer CropScience, Germany) [1]. From 2015 to 2018, the Magude project, designed to evaluate the feasibility of malaria elimination in Mozambique with available tools at the time, implemented annual rounds of IRS with DDT and Actellic® 300CS, on top of programmatically distributed insecticide treated nets (ITNs) and combined with mass drug administration and standard diagnosis and treatment [5].

Although great reductions in malaria incidence or prevalence were systematically observed during all the aforementioned initiatives, the fact that none of them managed to interrupt local malaria transmission [2, 6] calls for a thorough analysis to understand the limitations of the used interventions. IRS was the backbone for transmission reduction in all initiatives. Mozambique plans to continue using IRS to accelerate towards elimination in the south, and to reduce transmission in the highest burden areas and manage insecticide resistance throughout the country [7]. South African and Eswatini also continue to use IRS to progress towards malaria elimination [8]. IRS in general, continues to be a key vector control tool globally, not only for the control of malaria, but also for the control of several other vector-borne disease such as dengue, leishmaniasis and chagas disease [9]. A better understanding of the effectiveness of IRS campaigns will be critical to guide future malaria elimination efforts in Mozambique and southern Africa and efforts to control other vector-borne diseases.

IRS reduces malaria transmission by killing susceptible mosquitoes that rest indoors on sprayed walls, or by reducing indoor vector-host contact through its excito-repelling properties that prevent mosquito entry into houses or reduce the time they spend inside. Its effectiveness therefore depends on the resting and feeding behaviors of local malaria vectors [10], vector susceptibility to the IRS active ingredients [11], the IRS coverage that is achieved [12], the quality of spraying [13] and the residual efficacy of the IRS product over time [14]. IRS is considered to be most effective in areas where the local vectors rest indoors and are susceptible to the active ingredient of the IRS product. Its effectiveness increases with higher spray coverage as well as using active ingredients with a longer residual efficacy.

IRS campaigns are commonly evaluated by quantifying their operational coverage and -to a lesser extent—the product’s residual efficacy, but these indicators do not provide a complete picture of the potential effectiveness of an IRS campaign. Coverage is commonly reported as the percentage of houses or structures sprayed out of all those identified during IRS campaigns. Since some houses/structures may not be identified or are not accessible, this indicator can overestimate the actual coverage. Residual efficacy is often measured as the number of months during which mosquito mortality 24h post-exposure to a sprayed wall remains above 80% [15]. However, there is evidence that IRS leads to significant reduction in malaria prevalence (compared to no IRS) even after the mosquito mortality are below 80% [16, 17]. This may be linked to the delayed mosquito mortality induced by some IRS products (e.g. mortality 48 or 72h after exposure to the insecticide) [18, 19], or to other effects that sublethal exposure to IRS products may have on mosquitoes [20, 21]. In addition, efficacy measured through WHO cone bioassays, even when considering delayed mortalities, only reflects the duration of IRS efficacy on an individual sprayed wall. Since not all houses are sprayed during a campaign, a proportion of indoor resting mosquitos will be able to rest on unsprayed surfaces and hence will not be killed or affected. Furthermore, a product’s residual efficacy varies between different surface types [22–27] and as such, not all houses will have the same capacity to kill mosquitoes. Finally, IRS campaigns can take several months to be completed. Therefore, by the time the last houses are sprayed, the residual efficacy in the first sprayed houses would have partially waned off, affecting the overall ability of IRS to kill mosquitoes and hence the overall community protection of IRS [28]. These factors have not been systematically considered in the evaluation of IRS campaigns to-date but are likely to result in a lower realized IRS efficacy compared to estimates based on more frequently reported indicators.

In the present study, we examine the IRS campaigns conducted during the Magude project to understand their potential effectiveness and to identify gaps in the protection of IRS that may have jeopardized the interruption of malaria transmission in the district. We report the susceptibility of local vectors to deltamethrin, DDT, pirimiphos-methyl and bendiocarb, the operational and effective coverage of the IRS campaigns and the residual efficacy of Actellic® 300CS on cement and mud walls. We propose a new method to estimate the residual efficacy of IRS in a more realistic manner, which combines IRS coverage, the pace of spraying, the residual efficacy on different wall surfaces and the distribution of these wall types in the district into a new metric: the realized district-level IRS residual efficacy. We subsequently link this residual efficacy with seasonality of malaria transmission in the district to understand whether IRS effectively covered the high malaria transmission season during the Magude project.

## Materials and methods

### Study area

**Fig 1:**
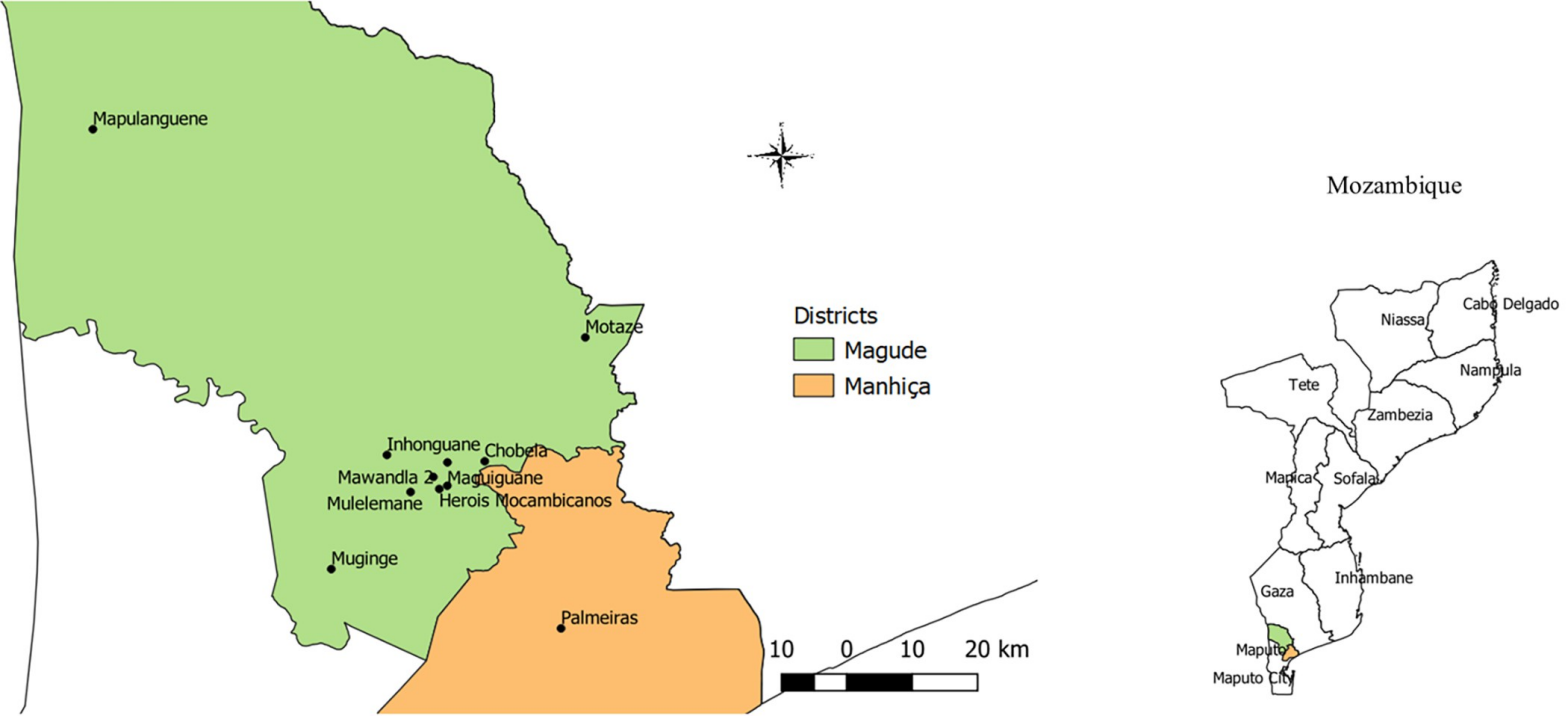
Map of the study areas. Villages/neighborhoods in Magude and Manhiça districts where adult mosquitoes were collected for insecticide resistance monitoring and/or WHO cone bioassays to evaluate the residual efficacy of Actellic® 300CS. The subnational administrative boundaries were obtained from the Humanitarian Data Exchange (https://data.humdata.org/dataset/cod-ab-moz) under a CC-BY-IGO license (https://data.humdata.org/faqs/licenses).

Magude district is a rural district in southern Mozambique that borders with South Africa (Kruger National Park) on the west (Fig 1). It covers approximately 6,961 km^2^ and, in 2015, had 48,448 residents and 4,133 non-residents divided over 10,965 households [29]. Detailed socio-demographic information on the district is provided elsewhere [6, 29]. Previous epidemiological analyses have shown that the high malaria transmission season in the district traditionally extends from November to April [29]. The main malaria vectors in southern Mozambique are *An*. *arabiensis* and *An*. *funestus s*.*s*. [3, 30–34]. In Magude, *An*. *arabiensis* was responsible for approximately 74% of all mosquito bites during the Magude project [35]. The district’s long IRS history is outlined in Table 1.

**Table 1:** History of indoor residual spraying (IRS) campaigns in Magude district.

Year (start IRS)*	Active ingredient	Coverage	Reference
2017	Pirimiphos-methyl	District level	[68]
2016	Pirimiphos-methyl	District level	[68]
2015	dichlorodiphenyltrichloroethane	District level	[68]
2014	Deltamethrin and DDT	Focal (Motaze)	[5] Personal communication NMCP
2013	Bendiocarb and Deltamethrin	District level	Personal communication NMCP
2012	No IRS conducted		
2011	DDT and Bendiocarb	District level	
2008–2010	Bendiocarb, Lambda-cyhalothrin and DDT	District level	
2007	Bendiocarb, K-otrine and DDT	District level	
2005, 2006	DDT and Bendiocarb	District level	

* IRS typically starts before the onset of the rainy season (August-October).

### Implementation of indoor residual spraying (IRS) in 2015, 2016 and 2017

District-wide IRS was implemented by Goodbye Malaria (GBM) in 2015 (3^rd^ of August to 7^th^ of November), 2016 (22^nd^ of August to 30^th^ November) and 2017 (21^st^ of August to 16^th^ December). In 2015, IRS was conducted with dichlorodiphenyltri-chloroethane (DDT) on thatched or mud-walled houses (47% of houses sprayed) and pirimiphos-methyl (Actellic® 300CS, Syngenta Crop Protection AG, Basel, Switzerland) on concrete-walled houses (53% of houses sprayed). In 2016 and 2017, only Actellic® 300CS was used in the IRS campaigns.

### Insecticide resistance testing

Insecticide susceptibility of *An*. *gambiae s*.*l*. and *An*. *funestus s*.*l*. to DDT (4%), bendiocarb (0.1%), deltamethrin (0.05%), and pirimiphos-methyl (0.25%) was assessed by means of standard WHO tube bioassays [36]. Wild blood-fed anopheline mosquitoes were collected indoors (from 6–10 am) using a mouth aspirator and a torch from April to September and in December of 2015, from February to August of 2016, from August to November of 2017 and from April to July of 2018. Mosquitoes were collected from the following villages/neighborhoods: Bairro 2000, Muginge, Motaze, Chobela, Maguiguane, Mulelemane, Mawandla 2, Mapulanguene, Herois Moçambicanos and Nhonguene (Fig 1). Collected *An*. *funestus s*.*l*. (1,042 adult females in 2015) and *An*. *gambiae s*.*l*. (1,024; 3,753; 508 and 412 adults females respectively in 2015, 2016, 2017 and 2018) mosquitoes were transferred to a climate-controlled insectary located in Manhiça district at the facilities of the Centro de Investigação em Saúde de Manhiça (CISM) (28±2°C, 75±5% RH, 12:12h day:night light cycle). Females from the same village were pooled into the same cage, given *ad libitum* access to a 10% dextrose solution (D-(+)-Glucose ≥99.5% (GC), Merck, Germany) and were allowed to oviposit. Larvae were reared in purified water (Elix® Advantage 3 Water Purification System and Millipore® Milli-DI) and fed with Tetramin® Baby fish food (Tetra Holding GmbH, Germany). WHO susceptibility tests were conducted with the 2–5 days old female adults that emerged. After exposure, mosquitoes were kept in the holding tubes for 24 hours with *ad libitum* access to a 10% dextrose solution (D-(+)-Glucose ≥99.5% (GC), Merck, Germany). Mortality was assessed 24-hrs post-exposure and mosquitoes stored individually on silica gel afterward (SiO2-Silica Gel Beads, Merck, Germany).

All tested mosquitoes were morphologically identified to either belonging to the *An*. *gambiae* complex or the *An*. *funestus* group using the dichotomous key of Coetzee [37]. A random subset of mosquitoes of each species group (approx. 22% of total sample size) were identified to species level by Polymerase Chain Reaction (PCR) as described by Scott *et al*. [38], and Koekemoer *et al*. [39], respectively.

### IRS operational and effective coverage, spray periods and reasons for not spraying

Data on the operational coverage of IRS (i.e. the number of houses and structures sprayed and found and the number of people living in sprayed households) was obtained from reports produced by GBM that implemented the campaigns. Data on IRS effective coverage (i.e. the number of households that reported receiving IRS) and on the reasons for households not being sprayed were assessed through structured questionnaires administered to Magude residents during the district-wide mass drug administration (MDA) campaigns in January 2016, after the first IRS campaign, and in February 2017, after the second IRS campaign. The date when households where sprayed was collected from the households of the randomly selected participants of each malaria prevalence cross-sectional survey conducted during the Magude project (May of 2017 and 2018). During these surveys, field workers copied the spray date from the sticker placed by the spray operators on one door of each sprayed household, when this was available. Details on MDAs and the demographic and health platform, their implementation periods and data collection forms, are provided elsewhere [6, 29].

### Monitoring the residual efficacy of Actellic® 300CS

The residual efficacy of Actellic® 300CS after the 2016 and 2017 campaign was evaluated through regular WHO standard cone bioassays conducted on the sprayed walls of a subset of nine cement and nine mud/clay houses, the two most common types of houses in the district [29]. Houses were selected from daily lists of sprayed houses. The number of houses was based on logistical feasibility. Neighboring unsprayed houses of the same wall types served as controls. During the 2016–2017 season, monitoring started 24h after IRS application and continued for a total of 12 months (August 2016 to September 2017). During the 2017–2018 season, monitoring started approx. one month after IRS application and continued for 10 months (November 2017 to August 2018). WHO cone bioassays were conducted in the same houses over the residual efficacy monitoring period. Residual efficacy was not evaluated after the 2015 campaign.

**Fig 2:**
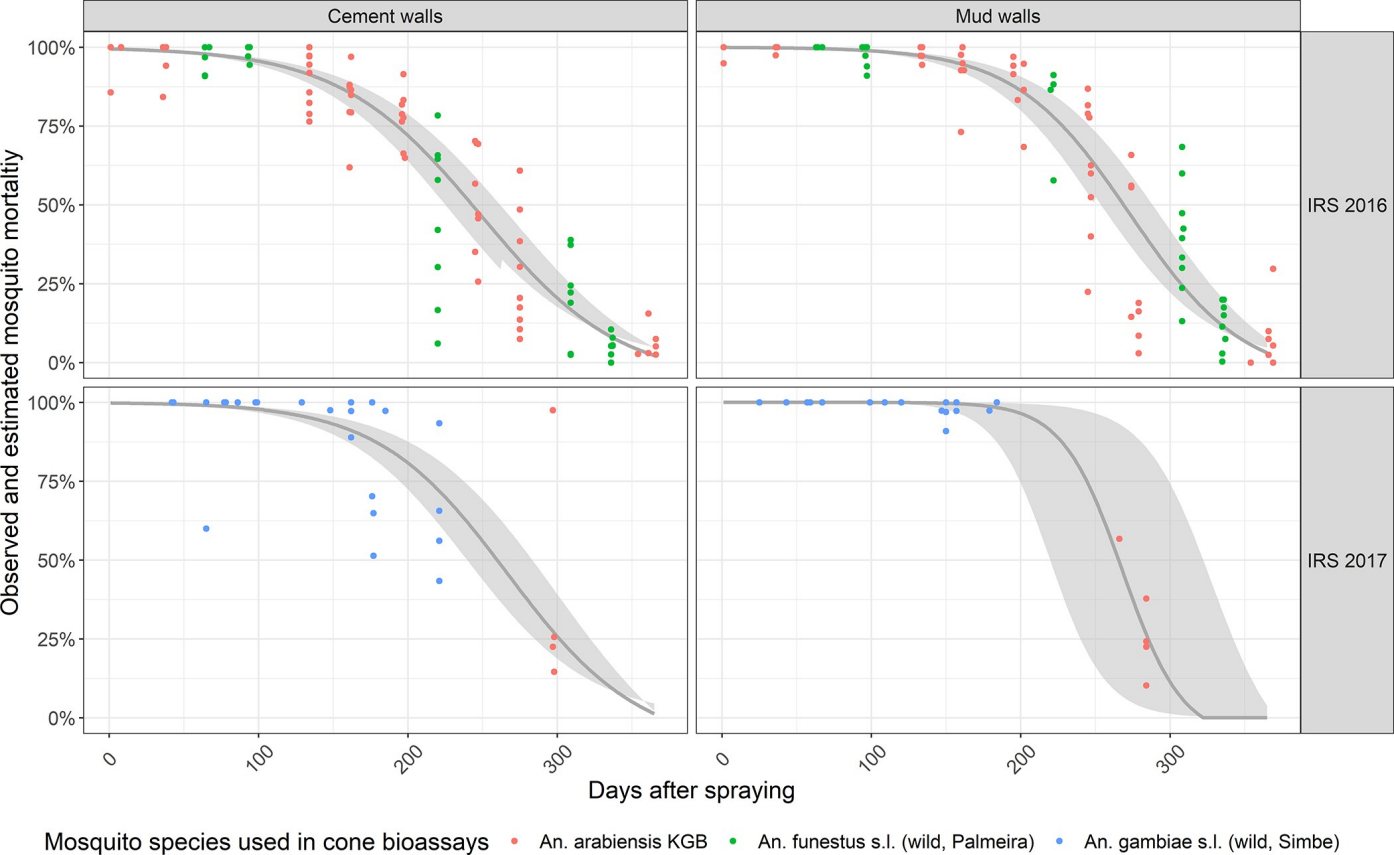
Residual efficacy of Actellic® 300CS in Magude on two different wall types after the 2016 and 2017 IRS campaigns in Magude district. Observed (point data, after Abbott’s correction) and estimated (lines) mosquito mortality 24h post-exposure to insecticide-treated mud/clay-plastered and cement walls. Point colors represent the species of mosquitoes used in cone bioassays at each point in time (*An*. *arabiensis* KGB colony mosquitoes, wild-caught *An*. *funestus s*.*l*. or wild-caught *An*. *gambiae s*.*l*.).

WHO standard cone bioassays were conducted using either mosquitoes from an insecticide susceptible *An*. *arabiensis* colony (KGB) or wild caught *An*. *funestus s*.*l*. or *An*. *gambiae s*.*l*.. The KGB strain originates from Kanyemba, Zimbabwe, and was colonized in 1975 and kept under standard insectary conditions as described by Hunt *et al*. [40] before a colony was started at CISM in 2015 in its climate-controlled insectary. Susceptibility of the colony to pirimiphos-methyl (the active ingredient in Actellic® 300CS) was confirmed using the WHO susceptibility bioassays [36] in July 2016 and June and July 2018 (S1 Table). When the number of mosquitoes from the susceptible KGB colony were insufficient to conduct WHO cone bioassays, either wild *An*. *funestus s*.*l*. collected in Palmeira, Manhiça district, or wild *An*. *gambiae s*.*l*. collected from Muginge and Simbe, Magude district, were used to conduct the WHO bone bioassays. Fig 2 shows the months when each of these types of mosquitoes were used. *An*. *funestus s*.*l*. and *An*. *gambiae s*.*l*. mosquitoes were collected indoors in the morning between 6–10 am. Specimens were obtained using a mouth aspirator and a torch. Female mosquitoes were selected and used in the WHO cone bioassays the following day. After the test, they were identified to species morphologically using a stereomicroscope and the dichotomous key of Coetzee [37]. The susceptibility of the *An*. *funestus s*.*l*. mosquito population from Palmeira and *An*. *gambiae s*.*l*. mosquito population from Magude to Actellic® 300CS was confirmed through several WHO susceptibility bioassays conducted during the study period (S2 Table). These tests were conducted with unfed 2–5 day old female offspring of the wild caught mosquitoes. Mosquito collection and rearing was done as described in the resistance monitoring section above.

In each house, WHO cone bioassays were conducted during the morning hours (6–10 am). WHO cones were positioned at four different heights (approx. 0.4m, 0.8m, 1.2m and 1.6m) arranged diagonally across a single wall. Ten 2–5 day-old unfed female mosquitoes susceptible to the insecticide sprayed were introduced in each cone and kept inside for 30 minutes [12, 15]. After this period, mosquito knock-down was recorded and the mosquitoes were transferred to paper cups and transported to a climate-controlled insectary with *ad libitum* access to a 10% dextrose solution (D-(+)-Glucose ≥99.5% (GC), Merck, Germany). Mortality among exposed and control mosquitoes was recorded 24h post-exposure and also 48h, 72h, 96h and 120h post-exposure to assess delayed mosquito mortality. After the 2016 IRS campaign, mortality 48h and 72h after exposure was only recorded from month 8 post-spraying (when 24h mortality fell below 80%) and 96h and 120h mortality from month 11 post-spraying. The dates, house type, mosquito species used, and results of individual bioassays are provided in S1 File.

### Data analysis

All analyses were conducted with R statistical software version 4.1.0. [41].

#### Vector resistance to insecticides

Mosquito mortality was assessed 24h after mosquito exposure to insecticide-treated or control papers and was calculated as the percentage of mosquitoes that died out of the total number of mosquitoes exposed. When control mortality was higher than 20%, the bioassay was discarded. When it was between 5% and 20%, the mortality of the exposed mosquitoes was corrected using Abbott’s formula [36]. Resistance status was defined according to WHO guidelines as: susceptibility (mortality 98–100%); suspected resistance (mortality 90–97%), and confirmed resistance (mortality below 90%) [36].

#### Operational and effective IRS coverage and reasons for not spraying

Operational house and structure-level coverage was calculated per IRS round based on the number of houses or structures sprayed out of those found during the campaign (as reported by the Goodbye Malaria). Population-level operational coverage was calculated as the number of people that were protected (as reported by the Goodbye Malaria) divided by the total number of residents in the district (as recorded through the census of the population and the demographic and health surveys [29]). Household-effective level coverage was calculated as the number of households that claimed receiving IRS of all the households for which spraying status was recorded during MDAs. Reasons for a household not being sprayed (as reported during the MDAs) are reported as frequencies.

#### Residual efficacy of Actellic® 300CS as measured through cone bioassays

Differences in mortality across cone heights were analyzed using Poisson regression models fitted using maximum likelihood (R package mixlm [42]), where Abbott’s adjusted mosquito mortality 24h post exposure was estimated by the cone height (lower, middle, upper) and wall surface (mud, cement), with the number of houses in which cone bioassays were conducted as the offset. This method was used because it allows to compare mortalities at different cone heights over time. Since there were no significant differences across cone heights (assessed at the 95% confidence level), data from individual cones was grouped for each house and test. The calculation of the district-level realized residual efficacy explained below requires daily estimates of mosquito mortality in WHO cone bioassays on different wall types. To estimate such daily mosquito mortalities with robust credible intervals, a logistic binomial Bayesian model was fitted to the mosquito mortalities observed in the WHO cone bioassays at each observation time post-exposure (24h, 48h, 72h, 96h and 120h), for each wall type and for mosquitoes exposed to treated and control walls separately. This method estimates daily mosquito mortality from the observed bioassay results at discrete points in time post-spraying. It simulates a sequence of random samples of mosquito mortalities from WHO cone bioassay that converge to the observed distribution of mosquito mortalities in the discrete WHO cone bioassays. In other words, it is a way to conduct a robust interpolation of observed bioassay results to obtain daily mosquito mortality values. A Hamiltonian Monte Carlo sampling methods was used and programmed using the R package RStan, the R interface to Stan programming language [43]. Four chains were initialized to assess the convergence of 1,000 iterations, the first 500 of each were discarded as burn in. The posterior distributions of parameters (4,000 iterations) and 90% Bayesian credible intervals were estimated, posterior checks were performed using R package shinystan (version 2.50.0) [44, 45] and visually confirmed to fit the data. Bioassays where control mortality 24h post-exposure was >20% were discarded from the analysis. Estimated daily mosquito mortality 24h post-exposure was corrected with Abbott’s formula when control mortality was > = 5% and < = 20% [15, 46]; estimated 48h, 72h, 96h and 120h mortalities post exposure were corrected when their respective control mosquito mortality was > = 5% but not discarded if mortality exceeded 20% to avoid losing data. We report the resulting mosquito mortalities 24h, 48h, 72h, 96h and 120h post exposure over time with their 95% confidence intervals and the number of days during which such mortalities remained above the WHO thresholds of 80% with their confidence intervals [15].

#### District-level realized IRS efficacy

Residual efficacy, as measured through WHO cone bioassays, represents the maximum mosquito killing efficacy of a sprayed wall over time. However, it does not represent the mosquito killing capacity of an IRS campaign in a sprayed area over time. This is due to the fact that: 1) efficacy is different for different types of walls and each area has a specific distribution of wall types; 2) by the time the last houses are sprayed during a campaign, the first sprayed houses may have started losing their efficacy, and 3) not all houses are sprayed. In order to obtain an estimate of the district-level realized IRS efficacy (i.e. the actual capacity of an IRS campaign to kill indoor resting mosquitoes at any given point in time since the beginning of the campaign), daily estimates of 24h-post-exposure mosquito mortality from the cone bioassays were adjusted by the distribution of wall types in the district, the pace of household spraying during the campaign (i.e. percentage of households actually sprayed at any given day after the campaign started, out of all households visited in the district at the end of the campaign) and the achieved effective IRS coverage. To do so, a weighted average of the estimated daily mosquito mortalities across wall types was calculated by giving cement houses a weight of 53% and mud/clay plastered houses a weight of 47%. These values are based on the proportion of houses of each type that were sprayed during the 2015 IRS campaign [6]. Since household wall types were not recorded during the malaria prevalence cross-sectional surveys or the 2016 and 2017 IRS campaigns, the weighted average mortality was assumed to represent the average residual efficacy of a household in the district. For each sprayed household, its daily IRS residual efficacy was calculated for 365 days from the time of spraying. Subsequently, for every day since the start of the campaign, the residual efficacy of each household with a spray date was summed and divided by the total number of houses sprayed for which the spray date was known. This represents the maximum daily residual efficacy that would have been achieved since the beginning of the campaign if all households would have been sprayed in the district. However, since a percentage of the households were not sprayed, the daily maximum residual efficacy was scaled to the percentage of households that were actually sprayed during the campaign. The result is the percentage of indoor resting mosquitoes that the IRS campaign could have killed in the district every day from the beginning of the campaign and is referred to here as the ‘realized district-level IRS residual efficacy’. The decay of this efficacy over time after the 2016 campaign is reported. WHO recommends reporting the number of weeks/months during which mosquito mortality 24h-post exposure stays above 80% for the evaluation of the residual action of insecticide impregnated surfaces [15]. Hence, this measure has been frequently reported across scientific literature. Although it is known that IRS continues to reduce malaria burden (compared to no IRS) beyond the point when mortality in mosquitoes exposed to spray walls falls below 80% [16], we report the number of days during which mosquito mortality remained equal to or greater than 80% (here called “optimal realized district-level IRS residual efficacy) to facilitate the comparison with results provided in other publications. Data was analyzed using R statistical software version 4.1.0. [41].

### Ethical considerations

Ethical approval for the monitoring of residual efficacy of IRS was obtained from Manhiça Health Research Centre Institutional Bioethics Committee for Health (CIBS-CISM/68/2015). Approval for monitoring insecticide resistance was obtained from the Manhiça Health Research Scientific Committee (CCI/135/Nov 2015). The household owner (>18 years old) where (i) mosquitoes were collected indoors for resistance monitoring or where (ii) the WHO cone assays were performed monthly, were informed about the purpose of the study in the local language (Xichangana or Portuguese) and gave their oral informed consent. They were free to withdraw from the study at any moment. All other studies from which data were drawn in the present study were approved by CISM’s institutional ethics committee, Hospital Clinic of Barcelona’s Ethics Committee, and the Mozambican Ministry of Health National Bioethics Committee. The study protocol to implement and evaluate the impact of MDAs was also approved by the pharmaceutical department of the Ministry of Health of Mozambique and registered as Clinical Trial NCT02914145. More details on the ethical consideration of the population census, household surveys, cross-sectional surveys and MDAs are provided elsewhere [6].

## Results

### Susceptibility status of *An*. *funestus s*.*l* and *An*. *gambiae s*.*l* and species composition

*Anopheles funestus s*.*l*. was only collected in sufficient numbers for susceptibility testing in 2015. *An*. *funestus s*.*l*. was susceptible to DDT, bendiocarb and pirimiphos-methyl, but resistant to deltamethrin (Table 2). Of the 22% of the *An*. *funestus s*.*l*. mosquitoes that were identified to species molecularly, the majority were either *An*. *funestus s*.*s*. (55.8%) or *An*. *parensis* (41.6%). Out of the 173 exposed and 93 control *An*. *funestus s*.*l*. mosquitoes used for resistance testing against deltamethrin, we identified to species 106 of the exposed and 65 of the control mosquitoes. Among the exposed mosquitoes, 40 were *Anopheles funestus s*.*s*. and 66 *An*. *parensis*. Among the control mosquitoes, 38 were *An*. *funestus s*.*s*. and 28 *An*. *parensis*. Among the exposed *Anopheles funestus s*.*s*., 17 died post-exposure (42.5%, n = 40), and among the exposed *An*. *parensis*, 58 died post-exposure (87.9%, n = 66). Differences between *An*. *funestus s*.*s*. and *An*. *parensis* mortalities were statistically significant χ^2^ = 22.641, df = 1, p<0.0001). After implementation of district-wide IRS with DDT and Actellic in 2015, *An*. *funestus s*.*l*. mosquitoes were no longer collected in sufficient numbers to evaluate whether resistance to DDT or pirimiphos-methyl emerged in this species after IRS.

**Table 2:** Insecticide susceptibility of F1 generation *An*. *funestus s*.*l*. and *An*. *gambiae s*.*l*. from Magude district, 2015–2018. Italics are used to indicate suspected resistance (mortality 90–97%); bold numbers indicate confirmed resistance (mortality below 90%).

		Bendiocarb 0.1%		DDT 4%		Deltamethrin 0.05%		Pirimiphos-methyl 0.25%	
		Percent mortality (n)		Percent mortality (n)		Percent mortality (n)		Percent mortality (n)	
		Treated	Control	Treated	Control	Treated	Control	Treated	Control
***An*. *gambiae s*.*l*.**									
2015	Bairro-2000	100 (197)	3.2 (94)	99.1 (134)	9.5 (63)	98.7 (98)	14.6 (48)	100 (112)	8.9 (45)
	Muginge			100 (86)	4.5 (44)			100 (97)	4.1 (49)
2016	Bairro-2000	*94*.*3* (122)	0 (74)	100 (166)	0 (94)	100 (88)	0 (62)	100 (140)	1.4 (71)
	Chobela	100 (116)	1.5 (67)	100 (224)	5.9 (119)	98.1 (214)	0.8 (124)	100 (113)	2.6 (70)
	Herois Mocambicanos	100 (102)	0 (41)						
	Maguiguane			100 (44)	13.6 (22)	100 (127)	0 (72)	100 (167)	7.5 (93)
	Mapulanguene			100 (97)	0 (48)				
	Mawandla 2			100 (100)	0 (49)			100 (49)	0 (24)
	Motaze			100(48)	0 (24)	100 (47)	0 (24)		
	Muginge			100 (100)	0 (49)	100 (98)	0 (50)		
	Mulelemane	100 (91)	4.2 (48)	100 (49)	0 (25)			100 (23)	0 (15)
	Nhongane			100 (96)	0 (48)			100 (96)	0 (44)
2017	Maguiguane	100 (21)	0 (10)	100 (45)	0 (24)	100 (112)	1.9 (53)	100 (67)	3.2 (31)
	Motaze	100 (20)	0 (10)			100 (56)	0 (28)	100 (14)	3.2 (31)
	Simbe	100 (78)	0 (30)			100 (78)	0 (30)	100 (70)	6.6. (30)
2018	Muginge			*97*.*4 (86)*	11.7 (51)	100 (100)	16 (50)	*95*.*4* (101)	12 (50)
2019	Muginge			100 (100)	0 (50)			100 (97)	2 (50)
***An*. *funestus s*.*l*.**									
2015	Bairro-2000	100 (72)	8.3 (60)	100 (37)	0 (38)	**67.6** (155)	8.3 (96)	100 (231)	8.3 (144)
	Muginge							100 (71)	0 (20)

Percentage indicates percent mortality 24h following 1h exposure to the insecticide; number between parentheses indicates the number of mosquitoes tested.

*An*. *gambiae s*.*l*. was susceptible to DDT, bendiocarb, pirimiphos-methyl and deltamethrin throughout the Magude project (Table 2). *An*. *gambiae s*.*l*. resistance to pirimiphos-methyl was suspected in Muginge in 2018, but its susceptibility to this insecticide was confirmed in the same village a year later (Table 2). Most (93%) of the identified *An*. *gambiae s*.*l*. mosquitoes were *An*. *arabiensis*.

### IRS operational and effective coverage, campaign duration and reasons for households not being spraying

In general, the IRS campaigns took place between the months of August and December and were completed within 3 to 4 months (S1 Fig). A summary of the campaigns and their outcomes is given in Table 3. The house and structure operational coverage (i.e. percentage of houses and structures sprayed out of those found during each campaign) was >90% for all campaigns. Population-level operational coverage (i.e. percentage of people living in sprayed household) was >86% in all campaigns. The household level effective coverage, as measured shortly after each IRS campaign, were 83% in January 2016 and 90% in February 2017 with little spatial heterogeneity [6]. This indicator was not assessed after the 2017 campaign.

**Table 3:** Coverage and duration of the IRS campaigns implemented during the Magude project.

	2015 campaign	2016 campaign	2017 campaign
**Period**	3rd August - 7th November	22nd August- 30th November	21st August- 16th December
**Campaign duration**	3 months + 4 days	3 months + 8 days	3 months + 25 days
**Household level-effective coverage** ^**1**^	83% (MDA2, Jan 2016)	89.7% (MDA4, Feb 2017)	ND
**- By administrative subdivision (MDA)**			
*Magude Sede*	81.3%	89.9%	ND
*Mahele*	91.4%	90.9%	ND
*Mapulanguene*	83.7%	86.2%	ND
*Motaze*	83.4%	90.0%	ND
*Panjane*	82.2%	85%	ND
**Population level operational coverage** ^**2**^	92.6%	86.1%	88.6%
**House-level operational coverage** ^**3**^	92.6%	94.5%	98.4%
**Structure-level operational coverage** ^**4**^	91.6%	92.6%	96.5%

1 Proportion of households sprayed of all households in Magude district. Results previously reported in [6].

2 Number of people that were protected (as reported by the Goodbye Malaria) divided by the total number of residents in the district.

3 Number of houses sprayed out of those found during the campaign (Results reported by Goodbye Malaria Initiative).

4 Number of structures sprayed out of those found during the campaign (Results reported by Goodbye Malaria Initiative).

ND: not determined.

The most commonly reported reason for a household not being sprayed was the fact that nobody was at home at the time the spray team visited the compound (51.5–64.1%), followed by the spray team not visiting the household (27.1–28.1%) and the household rejecting IRS (6.4–10.3%). In some cases (10.9–14.1%), the interviewee did not know why the household was not sprayed.

### Residual efficacy of Actellic® 300CS, 24h mortality

The residual efficacy of the 2016 IRS campaign (i.e. mosquito mortality >80% 24h after exposure) was estimated to be approximately 6 months (179 days, 95% CI: 163–196) on cement walls and 7 months (217 days, 95% CI: 199–236) on mud/clay plastered walls (Fig 2). The residual efficacy of the 2017 IRS campaign was estimated to be over 6.5 months (202 days, 95% CI: 182–224) on cement walls and close to 8 months (238 days, 95% CI: 194–292) on mud walls (Fig 2).

### Residual efficacy of Actellic® 300CS, delayed mortality

**Fig 3:**
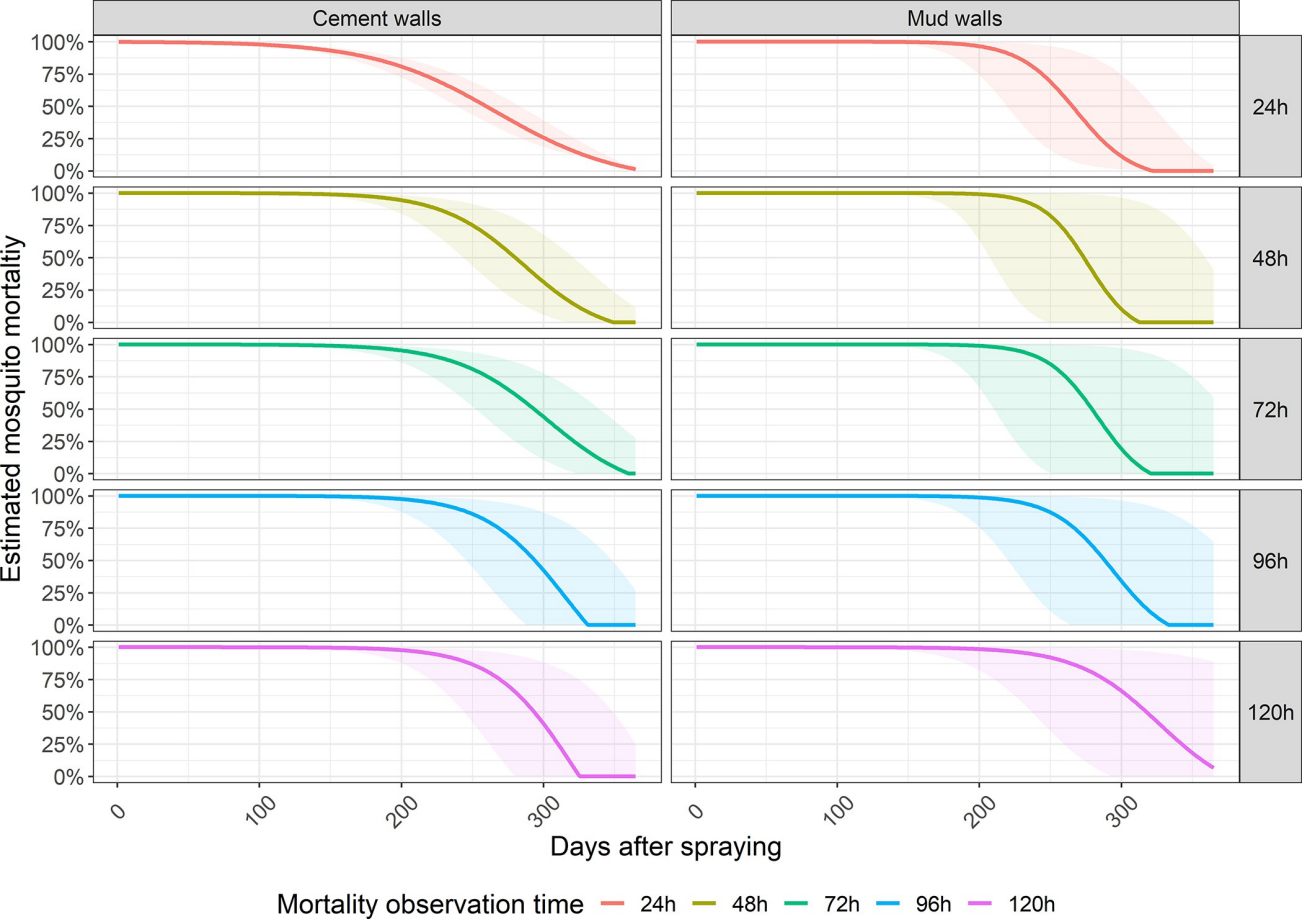
Estimated Abbott’s corrected mosquito mortalities 24h-120h after exposure to mud or cement walls sprayed with Actellic® 300CS during the 2017 campaign in Magude district.

Delayed residual efficacy could not be estimated for the 2016 IRS campaign as delayed mortalities were only assessed from month 8 post-spraying onwards. But observed Abbott’s corrected mosquito mortalities measured 72h post-exposure remained above 80% for 247 days on cement and 274 days on mud walls, 96h post-exposure mortality were already below 80% when it was measured for the first time (day 335) and 120h post-exposure mortality was above 80% for 235 days post-campaign in mud houses, but below 80% in cement houses at that same time point (Fig 3).

After the 2017 IRS campaign, estimated Abbott corrected mosquito mortality after exposure to treated mud walls was above 80% for an additional two weeks when 48h and 72h post-exposure mortality were considered, for approx. another 3 weeks when 96h post-exposure mortality was considered, and approximately another 1.5 months when 120h post exposure mortality was considered, compared to 24h mortality. In cement houses, 48h post-exposure mortality remained above 80% for an additional month and one week, 72h mortality for an additional month and two weeks, and 96h and 120h mortality for an additional two months, compared to 24h mortality data (Table 4).

**Table 4:** Duration of optimal IRS residual efficacy (i.e. mosquito mortality >80%) in mud and cement walls, as estimated through WHO cone bioassays and expressed in days.

	2016 campaign	2017 campaign				
**End point**	**24h**	**24h**	**48h**	**72h**	**96h**	**120h**
**Mud walls**	217 (199,236)	238 (194, 292)	253 (194, 335)	255 (189,344)	261 (195,346)	280 (203, >365)
**Cement walls**	179 (163,196)	202 (182,224)	242 (207, 280)	251 (213,295)	261 (213,314)	262 (213,317)

Mortality among control mosquitoes in WHO cone bioassays ranged from 0% to 11% 48h post-exposure, 2–14% 72h post-exposure, 2–27% 96h post-exposure and 3–29% 120h post-exposure.

### District-level realized IRS efficacy

**Fig 4:**
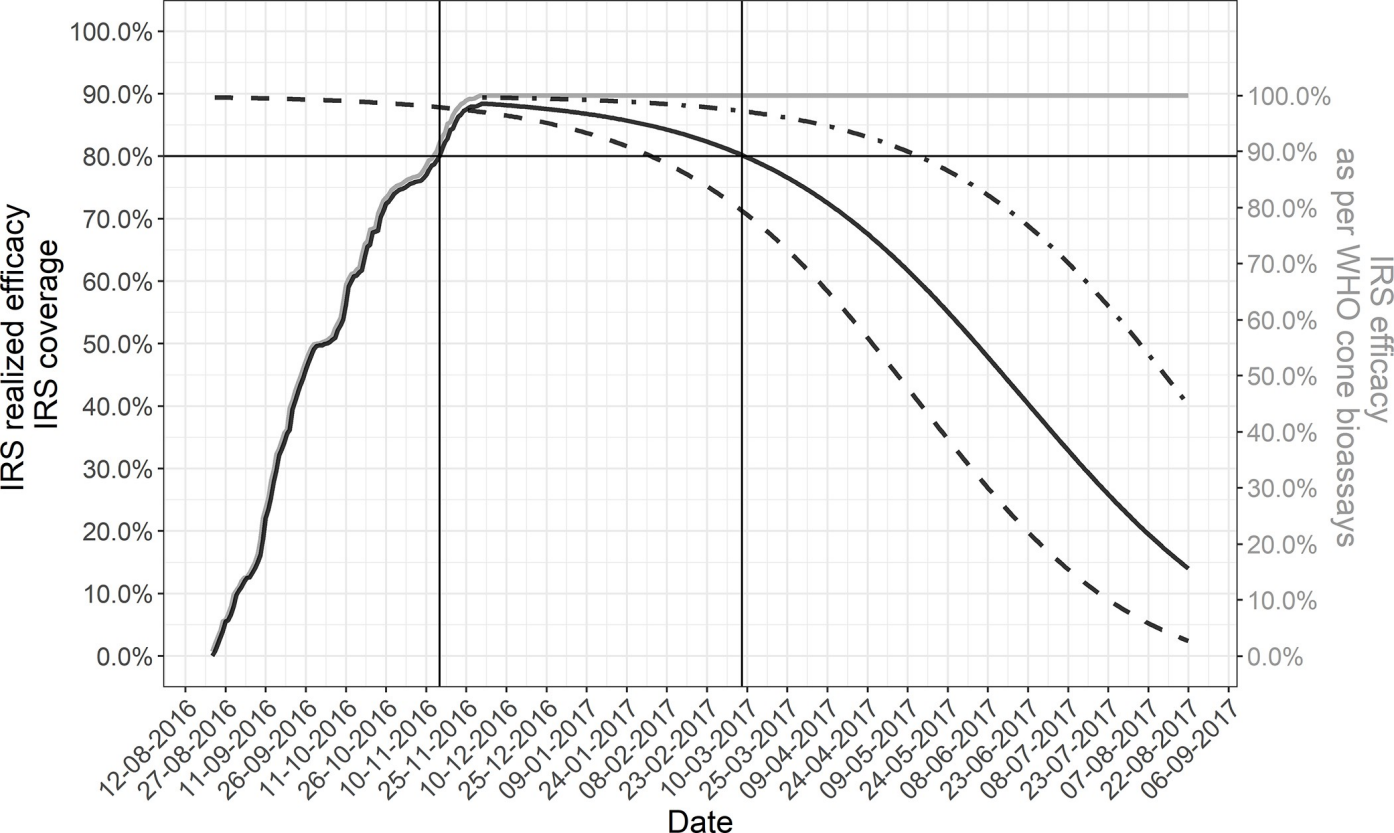
District-level IRS realized efficacy of the 2016 IRS campaign in Magude district. Grey solid line: IRS effective coverage (household level). Black solid line: Realized IRS residual efficacy in the district considering IRS coverage, the pace of spraying, residual efficacy in mud and cement walls and the distribution of these wall types in the district. To illustrate the effect of adjusting residual efficacy by pace of spraying, the dashed and dotted dashed lines represent how residual efficacy would have evolved if it started to decay at the beginning or the end of the campaign, respectively. Vertical lines mark the date when the campaign started to kill more than 80% of the mosquitoes resting indoors and when it started to kill less than 80% again.

Fig 4 shows the district-level realized efficacy overtime as a result of the 2016 IRS campaign in Magude district (i.e. the percentage of indoor resting mosquitoes that the IRS campaign could have killed on any given day after the campaign started).

**Fig 5:**
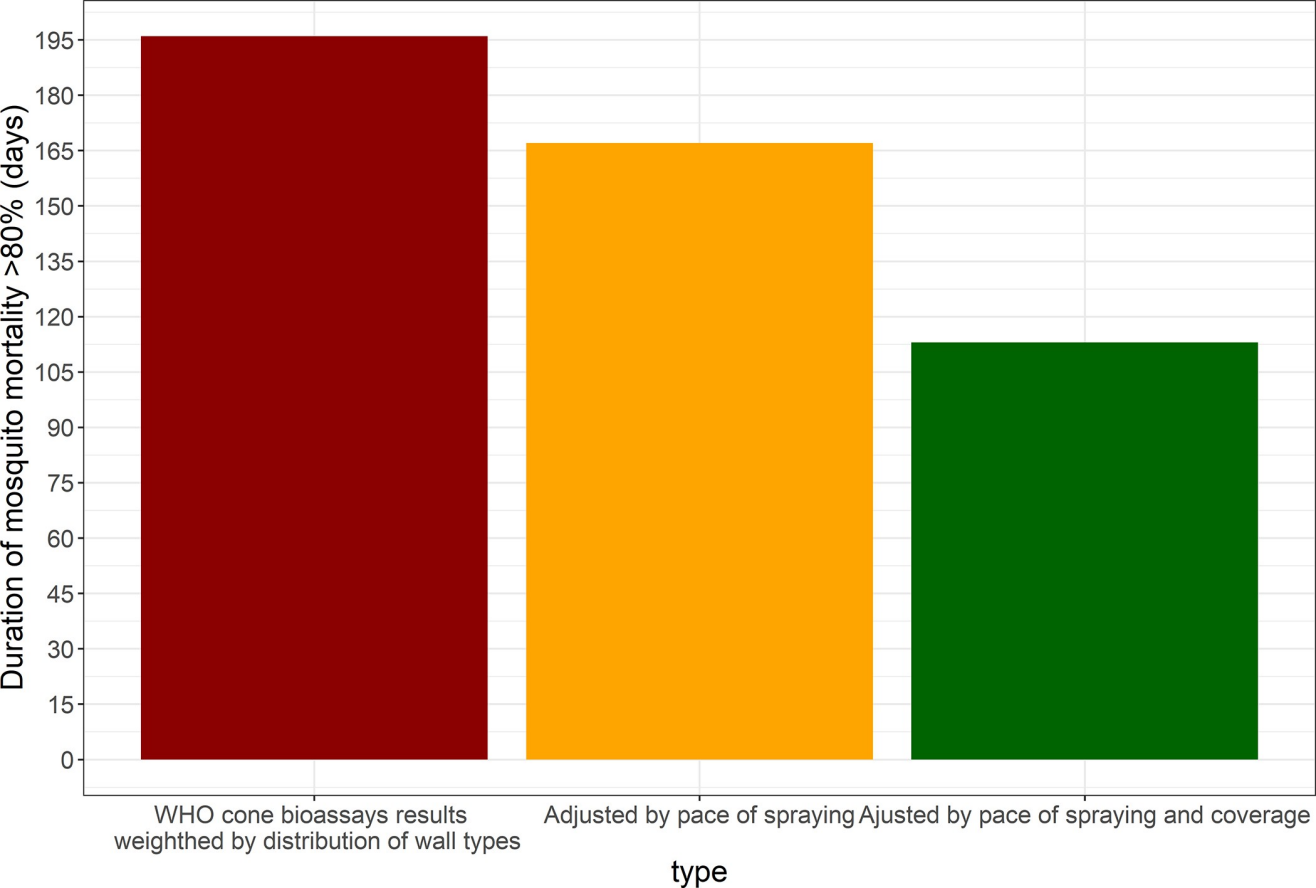
Reduction in the estimated duration of the 2016 IRS campaign residual efficacy after adjusting for wall type distribution, pace of household spraying and IRS coverage.

Considering the distribution of wall types in the district, the average duration of optimal residual efficacy of a sprayed wall in the district (measured as mosquito mortality 24-post exposure in cone bioassay remaining above 80%) was 6 months and 13 days (196 days, 95% CI: 179–213) after the 2016 IRS campaign (Fig 5), and 7 months and 8 days (222 days, 95% CI: 190–255) after the 2017 campaign. After correcting this for the pace of house spraying (shown in S1 Fig), if all households would have been sprayed, the district-level duration of optimal residual efficacy would have been 5 months and 15 days (167 days, 95% CI: 150–184) after the 2016 IRS campaign, which was achieved between 27^th^ October 2016 and 12^th^ April 2017 (Fig 5). After the 2017 IRS campaign it would have been 6 months and 9 days (191 days, 95% CI: 158–226) and achieved between 29^th^ October 2017 and 8^th^ May 2018.

Further adjustments for the IRS household-level effective coverage (i.e. percentage of household reported to be sprayed during the MDA campaigns of all district households), the optimal district-level realized efficacy after the 2016 campaign was shortened to 3 months and 20 days (113 days, 95% CI: 97–147) and achieved between 16^th^ November 2016 and the 9th of March 2017 (Fig 4). Household level effective coverage was not measured after the 2017 campaign and hence the district-level realized efficacy after this campaign could not be estimated.

## Discussion

The present study examined the IRS campaigns conducted during the Magude project to understand their effectiveness and gaps in protection that could help to explain why local malaria transmission was not interrupted in Magude and guide future malaria elimination efforts.

Local *An*. *funestus s*.*l*. and *An*. *gambiae s*.*l*. were susceptible to pirimiphos-methyl and DDT, the insecticides used for IRS in the 2015, 2016 and 2017 campaigns. *An*. *funestus s*.*l*. exhibited resistance to deltamethrin. Pyrethroid-resistance in *An*. *funestus s*.*l*. is wide-spread in southern Mozambique [31–33, 47]. However, in Magude district, *An*. *funestus s*.*l*. mortality after exposure to deltamethrin in 2015 (>67%) was substantially higher than mortalities observed in the neighboring districts of Chokwe (0% mortality in 2009 [33]) and Manhiça (33% mortality in 2009 [32]; 3–10% in 2014 [31]). These differences could be due to mosquito rearing or testing conditions, to the use of mosquitoes that were not truly representative of the local mosquito population or to true differences in the frequency of resistant mosquitoes between districts. A common limitation in all studies assessing discriminatory dose bioassay mortality using F_1_ offspring of wild caught female mosquitoes is how representative the tests are of the whole population. That is, if all offspring emerge from eggs laid by a few females, the survival at bioassay testing may simply reflect the specific phenotypes of those females rather than the distribution of phenotypes in the vector population. In our study, large amounts of blood-fed female mosquitoes were collected from several houses to increase the genetic diversity in the sample, but the percentage of adult females that laid eggs was not monitored. The previous studies in Manhiça and Chokwe have also not reported this critical information [31–33]. Ambient conditions are also known to affect resistance test results and could also explained the observed difference in resistance profiles [48]. However, differences could also reflect true differences in the vector population caused by natural barriers that prevent or limit gene-flow between mosquito populations, differences in selection pressure from historical agricultural practices or vector control interventions, variations in resistance status across species of the same complex [49] combined with inter-district differences in species distribution. It is worth noting that Manhiça, Chokwe and Magude districts all have large agricultural plantations where different insecticides may have been used over time to protect sugar cane or rice crops. The composition of the tested vector sample differed between Manhiça and Magude. In Magude, *An*. *funestus s*.*s*. accounted for 55.8% of the *An*. *funestus s*.*l*. mosquitoes, while in the Manhiça study it accounted for 95%. Although our assessment of the difference in the frequency of pyrethroid resistance among *An*. *funestus s*.*s*. and *An*. *parensis* is inconclusive due to the low number of *An*. *funestus s*.*s*. mosquitoes in the sample, our results would suggest a higher frequency of resistant individuals among *An*. *funestus s*.*s*. than among *An*. *parensis*. If these differences were confirmed, they could justify the inter-district differences in resistance observed. All in all, the inter-district differences in resistance to deltamethrin highlight the difficulty to extrapolate insecticide resistance results across areas. They should be further investigated molecularly and with large sample sizes collected in spatiotemporally diverse positions over a year to confirm that they are due to true differences in local vectors and not to artifacts during testing procedures. As new interventions targeting resistance mosquitoes emerge, such differences may be important to guide their deployment.

This is the third study that reports *An*. *parensis* resting on indoors walls [50, 51]. This is an important finding since *An*. *parensis* was recently incriminated as a malaria vector and implicated in residual malaria transmission in South Africa [52]. Although believed to be mainly zoophilic, *An*. *parensis* has been observed to bite humans outdoors [53]. During the Magude project, *An*. *parensis* accounted for 5.8% of residual vector bites [35] and was found feeding outdoors, indoors before people went to bed, and indoors while people were in bed, showing its potential to transmit malaria in different environments. Finding *An*. *parensis* resting indoors in the morning during the manual mosquito collections indicates that IRS could target a part of this vector population.

In contrast to *An*. *funestus s*.*l*., *An*. *arabiensis* from Magude district was susceptible to pyrethroids. This could indicate that this species manages to avoid or reduce exposure to insecticides through behavioral resistance. The ability of *An*. *arabiensis* to enter houses to blood feed but subsequently avoid contact with LLINs or IRS has been documented elsewhere [54, 55]. Given the number of large-scale agricultural plantations in and around Magude district, pyrethroids may have been used to protect crops in the area. If they were, the differences in the preferred breeding sites of *An*. *arabiensis*, *An*. *funestus s*.*s*. and *An*. *parensis* may have altered their exposure to pyrethroids, which could have contributed to the observed differences in their susceptibility to this class of insecticide [52].

The high proportion of houses and structures sprayed out of those found during the three IRS campaigns (>91%) suggests that IRS was well accepted by the population of Magude. This is further supported by the fact that rejection of IRS was only reported by 6–10% of people asked for the reason why their household were not sprayed. The population and household level coverage (>86% and >83%, respectively) indicate that the effective IRS coverage was high and above the WHO recommended coverage of 80% [56] during the Magude project. Although those two indicators were not measured after the 2017 campaign, effective coverage was likely to be equally high in this campaign as: i) the number of structures found during the campaign was similar, and; ii) the percentage of structures sprayed was higher compared to the two previous campaigns.

Interesting to note is the fact that the percentage of structures and houses sprayed of those found as reported by the IRS campaign was lower than the percentage of district households sprayed in all three IRS campaigns. As shown by the reasons for the households not being sprayed, this is likely due to the spray teams missing some households. This highlights the fact that the actual IRS coverage may be lower than the coverage reported after IRS campaigns, which could impact the efficacy of malaria control and elimination efforts.

Mosquito mortality 24h post-exposure in WHO cone bioassays remained above the WHO efficacy threshold of 80% [15] for over 7 months in mud houses, and around 6 months in cement houses. Although both KGB colony mosquitoes and wild collected mosquitoes were used in the cone bioassays, the pattern of residual efficacy decay seems to follow a similar and typical pattern [17]. Great differences in the duration of Actellic® 300CS’s residual efficacy have been observed across countries and wall types, with its efficacy ranging anywhere from 3 to 11 months [22–27, 57–59], and this tends to be true for other IRS products [60]. It has been argued that such differences could be related to the quality of spraying [26], differences in wall properties [61] (e.g., wall smoothness or coating used in different settings) or due to environmental conditions (e.g., temperature and humidity) [62] as well as potential differences in wall modifications post-spraying [28] that were not measured in the present study. Regardless of the reason, the variability in study results highlight the importance of measuring the residual efficacy of IRS products locally, to inform the selection of IRS products and identify the optimal time for IRS campaign implementation.

Killing malaria vectors before they can actually transmit malaria to humans (in the days between the moment they get infected to the moment they become infectious after the sporogonic cycle, or in the days between blood meals of already infectious mosquitoes) is expected to reduce malaria transmission [12]. In Magude, mosquito mortality 5 days post exposure to Actellic® 300CS extended optimal efficacy by between one and two months, depending on wall type, which is similar to previous observations in India [18]. This highlights the importance of assessing delayed mosquito mortalities to understand the real effect of IRS in reducing the ability of the vector to transmit malaria. However, the high mortality among control replicates observed at 48h, 72h, 96 and 120h time points indicates that a broader discussion is needed to identify the best methods to estimate IRS induced delayed mosquito mortalities.

Traditionally, the potential impact of an IRS campaign has been assessed by reporting operational IRS coverage and a product’s residual efficacy (as measured by WHO cone bioassay) separately [12, 15]. But this provides incomplete information on the true potential mosquito killing effect of an IRS campaign. First, it does not take into account that houses are sprayed gradually and hence, residual efficacy does not decay in all houses at equal pace from the start (or end) of the campaign. Secondly, differences in residual efficacy across wall types implies that some houses have a higher mosquito killing capacity than others. As a result, some houses will be more effective at killing indoor resting mosquitoes than others at any given point in time during and after each campaign. Thirdly, unsprayed houses will provide surfaces for mosquitoes to rest on without being killed, reducing the overall capacity of the IRS campaign to kill indoor resting mosquitoes in the targeted area. By combining these factors, a more realistic metric of IRS efficacy, the “district-level realized IRS efficacy”, is presented here. Surprisingly, this new metric shows that the optimal realized district-level efficacy of the 2016 IRS campaign (with Actellic® 300CS) was 3 months and 20 days, almost 3 months shorter than the optimal residual efficacy measured through standard WHO cone bioassays alone. Based on WHO cone bioassay data alone, one would assume that the 2016 IRS campaign effectively covered the entire high malaria transmission season, but the realized efficacy was achieved mid-November (shortly after the start of the rainy season and a month and half before the high malaria incidence season) and lost early March (almost two months before the traditional high malaria transmission season ended). Although delayed mortality may extend the duration of the realized efficacy for an additional month or so, the rapid decrease in efficacy towards April may leave communities less protected at the end of the high malaria transmission season, a time when the effect of MDA had already faded away. This could explain the annual increases in malaria incidence observed during April, May and June throughout the Magude project [6]. Rains are still frequent and intense during those months [29], which could have created adequate conditions for vector populations to proliferate and drive transmission in the absence of effective IRS.

To identify strategies that could have closed this gap, it is crucial to understand the behavior of local vectors, as the effectiveness of vector control interventions depend upon them. The main malaria vector species during the Magude was *An*. *arabiensis* which accounted for 74% of all human exposure to vector bites [35]. *An*. *arabiensis* is known for the plasticity of its behaviors. It can feed on animals or humans, depending on host availability and feed indoors or outdoors, at dusk, dawn or during the night depending on the location of its hosts [54, 63]. It has been observed to rest indoors when its hosts are primarily indoors [64], but to rest both indoors and outdoors when its hosts are outdoors [65]. As said before, *An*. *arabiensis* is known for its ability to avoid contact with vector control interventions [54, 55] and has been found to exhibit outdoor resting tendencies following the application of IRS or deployment of ITNs [66, 67]. Given the variation of *An*. *arabiensis* across areas upon host availability and local situation, an evaluation of its local behaviors is necessary to understand the effect that different vector control interventions could have on the local population of this species. In our study, most of the mosquitoes that were collected indoors during the manual collections for resistance monitoring purposes, both before and after the implementation of IRS campaigns, were *An*. *arabiensis*. Although we did not measure how frequently this vector rests indoors (compared to outdoors) in Magude, the fact that *An*. *arabiensis* were found resting indoors combined with the high IRS coverage suggests that IRS controlled *An*. *arabiensis* partially but not fully. In addition, IRS is likely to have controlled *An*. *funestus s*.*s*., known to be a major vector in the region, as this vector species was no longer found in indoor collections for resistance monitoring after the implementation of the first IRS campaign. These facts suggest that IRS was effective, at least to some extent, at controlling the main local vectors during the Magude project and leads us to conclude that the use of a longer-lasting IRS product would have contributed to further reductions in malaria transmission.

Our proposed new methodology to estimate the IRS residual efficacy in a more realistic manner has some limitations: it requires 1) knowing the distribution of different house types in the district, which is information that is often not available unless a census of the population has been conducted recently, 2) understanding the different residual efficacies on different wall types, which is often not assessed in programmatic settings or only in a few geographic locations, and 3) knowing the pace of spraying (i.e. the proportion of structures sprayed on any given day out of all structures sprayed during the campaign), information that is often not reported after IRS campaigns but may be collected as part of the campaign monitoring process. One could omit information on differences in residual efficacies between wall types, and use a simplified version of our proposed realized IRS residual efficacy, but we argue that IRS coverage and the pace of spraying will be important indicators to better understand the efficacy of IRS campaigns.

Beyond shedding light onto the reasons why malaria transmission was not interrupted during the Magude project, this study highlights the need to rethink the data and indicators used to evaluate the potential effectiveness of IRS campaigns. The large differences in residual efficacy estimates obtained through WHO cone bioassays compared to those obtained by considering IRS coverage, the pace of spraying, the residual efficacy on different wall types and the distribution of such wall types in the district, shows the need to improve current methods to estimate IRS efficacy. Finally, the impact that the pace of household spraying had in determining the time when IRS campaigns reached optimal efficacy, highlights the need to evaluate different IRS implementation strategies to design the most effective IRS campaigns.

## Conclusion

The IRS campaigns implemented during the Magude project achieved high coverage and acceptability. However, its realized residual efficacy considering IRS coverage, the pace of spraying, residual efficacy on different wall types and distribution of such wall types in the district, fell short to provide optimal protection during the entire high malaria transmission season, which could be one of the reasons why local malaria elimination was not achieved. The use of a longer-lasting IRS product could have contributed to further reducing malaria transmission by increasing the protection provided during the final months of the high transmission season. An accurate estimation of IRS residual efficacy and an evaluation of vector behaviors and insecticide resistance is critical to select IRS products and to inform the overall design of vector control strategies. Countries should consider more realistic indicators, such as the realized IRS efficacy proposed here, to obtain more accurate estimates on the efficacy of their IRS campaigns.

## Supporting information

S1 Fig Pace of household spraying during the 2016 and 2017 IRS campaigns. (TIFF)

S1 Table Susceptibility of CISMs *An*. *arabiensis* KGB colony to pirimiphos-methyl. (DOCX)

S2 Table Susceptibility of F1 generation of wild-caught *An*. *funestus s*.*l*. and *An*. *gambiae s*.*l*. to pirimiphos-methyl. (DOCX)

S1 File Raw uncorrected mortality data for mosquitoes from all WHO cone bioassays conducted in Magude district from 2016 to 2018. (XLSX)

S2 File Dates on which households received IRS during the 2016 and 2017 campaigns in Magude, as recorded for those households of randomly selected participants in the malaria prevalence cross-sectional surveys in May of 2017 and 2018. (XLSX)
