# Supplementary figures and images for: The realized efficacy of indoor residual spraying campaigns falls quickly below the recommended WHO threshold when coverage, pace of spraying and residual efficacy on different wall types are considered
Source: PLoS One. 2022 Oct 3;17(10):e0272655. doi: 10.1371/journal.pone.0272655 (PMC9529131; doi:10.1371/journal.pone.0272655)

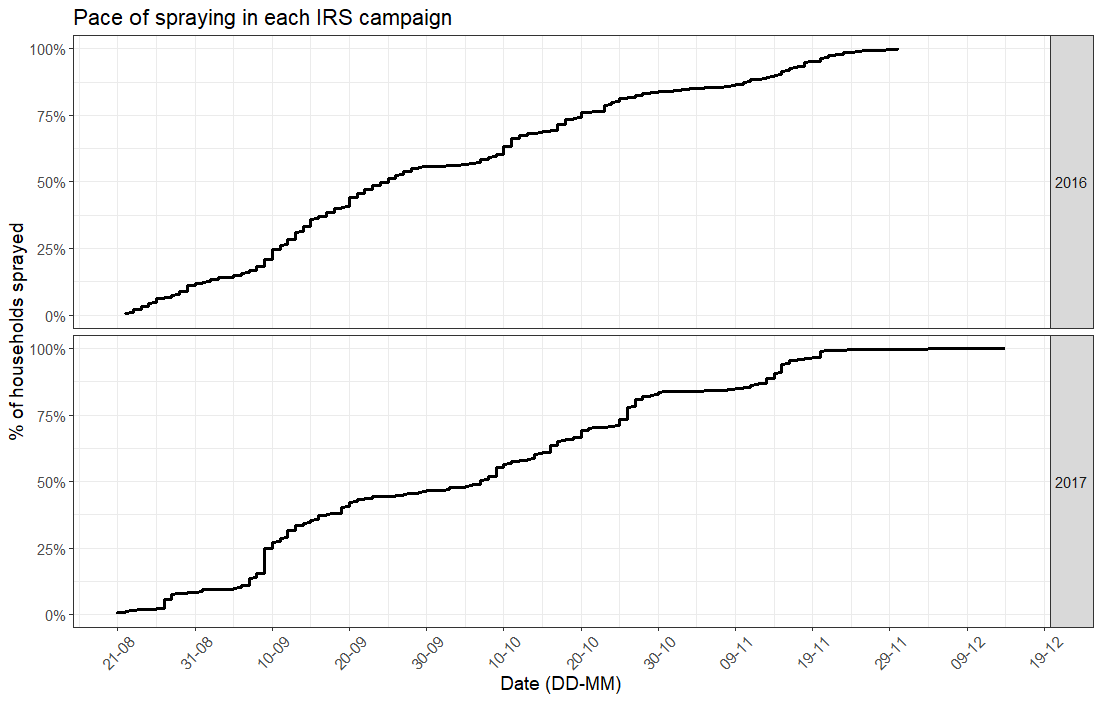

Supplement: S1 Fig — (TIFF) [file pone.0272655.s001.tiff]
