# Supplementary material for: The realized efficacy of indoor residual spraying campaigns falls quickly below the recommended WHO threshold when coverage, pace of spraying and residual efficacy on different wall types are considered
Source: PLoS One. 2022 Oct 3;17(10):e0272655. doi: 10.1371/journal.pone.0272655 (PMC9529131; doi:10.1371/journal.pone.0272655)
Supplement: S1 Table — (DOCX) [file pone.0272655.s002.docx]

**S1 Table. Susceptibility of CISMs *An. arabiensis* KGB colony to pirimiphos-methyl**

| **Test date** | **Mortality in controls** | **Abbott’s adjusted Mortality in exposed (%)** |
| --- | --- | --- |
| 27.07.16 | 0 (20) | 100(36) |
| 07.06.18 | 0 (51) | 98.7(76) |
| 18.07.18 | 7.3(41) | 98.3 (65) |
