# Supplementary material for: The realized efficacy of indoor residual spraying campaigns falls quickly below the recommended WHO threshold when coverage, pace of spraying and residual efficacy on different wall types are considered
Source: PLoS One. 2022 Oct 3;17(10):e0272655. doi: 10.1371/journal.pone.0272655 (PMC9529131; doi:10.1371/journal.pone.0272655)
Supplement: S2 Table — (DOCX) [file pone.0272655.s003.docx]

**S2 Table.** **Susceptibility of F1 generation of wild-caught *An. funestus s.l.* and *An. gambiae s.l.* to pirimiphos-methyl**.

| **Species** | **Locality** | **Test date** | **Mortality in controls (%)** | **Mortality in exposed (%)** |
| --- | --- | --- | --- | --- |
| *An. funestus s.l.* | Palmeira, Manhiça district | 19.05.2016 | 0 (22) | 100 (44) |
|  |  | 21.07.2016 | 0 (30) | 100 (56) |
|  |  | 29.08.2017 | 4.2 (24) | 100 (45) |
| *An. gambiae s.l.* | Muginge/Simbe, Magude district | 14.12.2017 | 6.7 (30) | 100 (70) |
